# Structure of the transmembrane protein 2 (TMEM2) ectodomain and its apparent lack of hyaluronidase activity

**DOI:** 10.12688/wellcomeopenres.18937.2

**Published:** 2023-05-02

**Authors:** Muyuan Niu, Molly McGrath, Douglas Sammon, Scott Gardner, Rhodri Marc Morgan, Antonio Di Maio, Yan Liu, Doryen Bubeck, Erhard Hohenester

**Affiliations:** 1 Department of Life Sciences, Imperial College London, London, SW7 2AZ, UK; 2 Glycosciences Laboratory, Department of Metabolism, Digestion and Reproduction, Faculty of Medicine, Imperial College London, London, England, W12 0NN, UK

**Keywords:** Glycosaminoglycan, hyaluronidase, X-ray crystallography, parallel β-helix, lectin

## Abstract

**Background: **Hyaluronic acid (HA) is a major polysaccharide component of the extracellular matrix. HA has essential functions in tissue architecture and the regulation of cell behaviour. HA turnover needs to be finely balanced. Increased HA degradation is associated with cancer, inflammation, and other pathological situations. Transmembrane protein 2 (TMEM2) is a cell surface protein that has been reported to degrade HA into ~5 kDa fragments and play an essential role in systemic HA turnover.

**Methods:** We produced the soluble TMEM2 ectodomain (residues 106-1383; sTMEM2) in human embryonic kidney cells (HEK293) and determined its structure using X-ray crystallography. We tested sTMEM2 hyaluronidase activity using fluorescently labelled HA and size fractionation of reaction products. We tested HA binding in solution and using a glycan microarray.

**Results:** Our crystal structure of sTMEM2 confirms a remarkably accurate prediction by AlphaFold. sTMEM2 contains a parallel β-helix typical of other polysaccharide-degrading enzymes, but an active site cannot be assigned with confidence. A lectin-like domain is inserted into the β-helix and predicted to be functional in carbohydrate binding. A second lectin-like domain at the C-terminus is unlikely to bind carbohydrates. We did not observe HA binding in two assay formats, suggesting a modest affinity at best. Unexpectedly, we were unable to observe any HA degradation by sTMEM2. Our negative results set an upper limit for *k*_cat_ of approximately 10 ^-5^ min ^-1^.

**Conclusions:** Although sTMEM2 contains domain types consistent with its suggested role in TMEM2 degradation, its hyaluronidase activity was undetectable. HA degradation by TMEM2 may require additional proteins and/or localisation at the cell surface.

## Introduction

Hyaluronic acid (HA), also called hyaluronan, is a polysaccharide consisting of alternating glucuronic acid (GlcA) and N-acetylglucosamine (GlcNAc) sugars, linked [GlcA-β1,3-GlcNAc-β1,4] _n_^1^. It is the only glycosaminoglycan that exists as a free polysaccharide; all other glycosaminoglycans, such as heparan sulphate or chondroitin sulphate, are attached to core proteins to form proteoglycans. HA is synthesised by a plasma membrane-embedded processive enzyme, which produces chains of molecular mass >1 MDa (high-molecular-weight HA, HMW-HA). HMW-HA retains a large amount of water and forms gel-like networks with high viscoelasticity, which makes it essential for tissue hydration and biomechanics. HMW-HA is anti-inflammatory, anti-proliferative, and anti-angiogenic ^1, 2^. In contrast, HA fragments derived from HMW-HA degradation often have opposite biological activities, contributing to a range of pathological conditions ^1, 2^. Therefore, the enzymes involved in HA turnover are of considerable interest.

The human genome encodes six members of the HYAL (hyaluronidase) family. HYALs are generally associated with acidic intracellular compartments such as the lysosome and sperm acrosome, but some members also exist as glycsoylphosphatidylinositol-anchored proteins at the cell surface where they may contribute to extracellular HA degradation ^3^. HYAL1 is considered to be the major lysosomal hyaluronidase. Recently, two homologous proteins that are not members of the HYAL family have been implicated in extracellular HA degradation: CEMIP (cell migration-inducing and hyaluronan-binding protein, also called KIAA1199 or HYBID) and TMEM2 (transmembrane protein 2). The two proteins share the same domain structure (see below) but differ in one important respect: CEMIP is a secreted protein, whereas TMEM2 is a type II transmembrane protein in the plasma membrane ^3^. CEMIP was found to be required for HA degradation in normal skin fibroblasts, and transfection with CEMIP endowed other cell types with HA-degrading capability ^4^.

Interestingly, active CEMIP appeared to be associated with clathrin-coated pits, and purified soluble CEMIP had no hyaluronidase activity. TMEM2 was shown to degrade HMW-HA into 5 kDa fragments in a cell contact and Ca ^2+^-dependent manner ^5^. Unlike in the case of CEMIP, a soluble version of TMEM2 had hyaluronidase activity, allowing a pH optimum of 6-8 to be determined. Subsequent studies showed that TMEM2 is essential for systemic HA turnover ^6^ and that it regulates cell adhesion and migration via HA degradation at focal adhesion sites ^7^. Interest in TMEM2 was further raised by a screen in *C. elegans* that identified TMEM2 as a promotor of endoplasmic reticulum homeostasis and longevity ^8^.

We set out to characterise TMEM2 structurally and enzymologically. Prior to the structure prediction of the human proteome by AlphaFold ^9^, the domain structure of TMEM2 was assigned only incompletely. Prediction by Phyre2 ^10^ identified the two lectin-like domains and a central β-helix. A G8 domain had been assigned at the N-terminus by a previous bioinformatic study ^11^, but was not picked up by Phyre2 because of the lack of structural information. Here, we describe the crystal structure of the entire TMEM2 ectodomain and our unsuccessful efforts to demonstrate its hyaluronidase activity. Together with other recent findings, our study should prompt a fresh look into the mechanism of TMEM2-mediated HA degradation.

## Methods

### Expression vector

DNA encoding the human TMEM2 ectodomain (soluble TMEM2, sTMEM2) was assembled from two partial cDNA clones (IMAGE clones 9021641 and 9053037; Horizon Discovery) using strand overlap extension polymerase chain reaction (PCR). The PCR product was cloned into the N-His-TEV-pCEP vector ^12^ using *Nhe*I and *Not*I. The correct sequence was verified by DNA sequencing. The vector encodes a protein consisting of the basement membrane protein 40 signal peptide, a hexahistidine tag, a tobacco etch virus protease cleavage site, and TMEM2 residues 106-1383 (KYAPDE...QASKAH). After cleavage of the signal peptide, the following vector-derived sequence remains at the N-terminus of secreted sTMEM2: APLVHHHHHHALDENLYFQGALA.

### Protein production

sTMEM2 was produced in Expi293 cells maintained in FreeStyle 293 medium (Thermo Fisher Scientific, catalogue number A14635) at 37°C and 8% CO _2_ in shaking flasks (125 rpm). The cells were transfected at a density of 10 ^6^ cells/ml using polyethylenimine (PEI MAX 40000; Polysciences) and a DNA:PEI ratio of 1:3 (1 μg of DNA/ml of cell culture). Five days after transfection the medium was harvested and the cells were pelleted by centrifugation at 500 g. The supernatant was passed through a 0.45 μm cellulose acetate filter (Sartorius) and loaded onto a 1-ml HisTrap column (Cytiva) using an ÄKTA pure chromatography system. The column was washed with 50 mM HEPES, 150 mM NaCl, pH 7.5 and the bound protein eluted in wash buffer containing 500 mM imidazole. Fractions containing protein were concentrated to 0.5 ml using a Vivaspin 30,000 MWCO centrifugal filter (Sartorius). The concentrated sample was loaded onto a Superdex 200 increase 10/300 column (Cytiva) and eluted in 50 mM HEPES, 125 mM NaCl, 2 mM CaCl _2_, pH 7.5. sTMEM2 eluted in a symmetric peak at 11.4 ml. The peak fractions were pooled, concentrated to 3 mg/ml, and snap-frozen in liquid nitrogen in 50 μl aliquots. The final yield was 8 mg of sTMEM2 protein from 1 litre of cell culture medium.

### Crystal structure determination

Snap-frozen purified sTMEM2 was thawed and rerun on a Superdex 200 increase 10/300 column (Cytiva). Peak fractions were concentrated to 9 mg/ml and a range of commercial crystallisation screens were set up using a Mosquito nanolitre liquid handler (STP Labtech). Large single crystals were obtained at room temperature using 0.1 M sodium acetate, pH 4.5, 30% polyethylene glycol 3000 as precipitant. The crystals were frozen in liquid nitrogen using precipitant solution supplemented with 20% ethylene glycol as cryoprotectant. Diffraction data were collected on beamline I04 at the Diamond Light Source (λ = 0.9795 Å) and processed using the XIA2 DIALS pipeline (version 3.dev.661-g1a4ae04e6) ^13^. The structure was solved by molecular replacement using PHENIX (version 1.18rc1_3769) ^14^ and AlphaFold 2.0 model Q9UHN6 as search model ^9^. Manual rebuilding and refinement were done using COOT (version 0.8.9.2) ^15^ and PHENIX. A conservative refinement protocol using a single B-factor per residue gave the same *R*_free_ as models with more parameters. Crystallographic data are summarised in Table 1. Structural comparisons were done using the DALI server ^16^. All software used in the study are freely available to academic users.

**Table 1.** Crystallographic statistics.

Data collection	
Resolution range (Å)	92.38–3.50 (3.56–3.50)
Space group	*P*4 _2_2 _1_2
Unit cell: *a*, *b*, *c* (Å)	184.76, 184.76, 105.40
Unique reflections	23570 (1159)
Multiplicity	26.8 (28.3)
Completeness (%)	100.0 (100.0)
Mean *I*/σ( *I*)	7.1 (0.5)
*CC*_1/2_	0.997 (0.424)
*R*_pim_	0.073 (0.871)
**Refinement**	
Non-hydrogen atoms Protein Glycan Ion	9992 333 1 Ni ^2+^, 1 Cl ^-^
*R*_work_/ *R*_free_	0.265/0.319
Root-mean-square deviations Bonds (Å) Angles (°)	0.004 0.638
Ramachandran plot Favoured (%) Allowed (%) Outliers (%)	90.1 9.2 0.7

### Enzyme activity assays

Fluorescein-labelled high-molecular-weight HA (HMW-HA; average molecular mass, 800 kDa; 4-6 mol-% substitution; Carbosynth) was incubated for 18 h at 37°C with sTMEM2 or hyaluronidase from bovine testes (750-3,000 U/mg; Sigma-Aldrich, H3884) in 100 mM MES, pH 6.0, 1 mM CaCl _2_ (total reaction volume, 1 ml). The HMW-HA concentrations ranged from 10 to 250 μg/ml, and protein concentrations ranged from 10 to 100 μg/ml. The reaction products were size-fractionated on a HiPrep 16/60 Sephacryl S200 HR column (Cytiva) with phosphate-buffered saline as running buffer. The useful separation range of this column for dextran standards is quoted as 1-80 kDa by the manufacturer. A fluorescein-labelled HA 22-mer (5 kDa, Iduron) was used as a reference sample. Aliquots of each fraction were transferred into black 96-well plates (Greiner Bio-One) and fluorescence was measured using a Tecan Spark microplate reader (excitation, 485 nm; emission, 535 nm). In a separate set of experiments, 0.1 μg/ml HMW-HA was incubated with 100 μg/ml of sTMEM2 or sperm hyaluronidase. The reaction products were passed through a Vivaspin 50,000 MWCO centrifugal filter (Sartorius) and the fluorescence in the filtrate measured as described above. The mock experiment contained 100 μg/ml of an irrelevant protein that is not known to bind or cleave HA (Endo180 D1-4 ^17^).

### Circular dichroism spectroscopy

sTMEM2 at a concentration of 0.12 mg/ml in 50 mM phosphate buffer, pH 6.0 was incubated overnight at 4°C and 37°C. Spectra were recorded on a Chirascan CD spectrometer (Applied Photophysics) in a 1-mm path length quartz cuvette at 20°C, and corrected for buffer signals.

### HA interaction assay

 sTMEM2 (1 mg/ml) was incubated with unlabelled HMW-HA (200 μg/ml; average molecular mass, >950 kDa; R&D Systems) for 30 min at room temperature in 50 mM HEPES, 125 mM NaCl, 2 mM CaCl _2_, pH 7.5 (total volume, 200 μl). The mixture was loaded onto a Superdex 200 increase 10/300 column (Cytiva) and eluted in the same buffer. The control experiment was done in the same way, but without HA.

### Glycan microarray

Microarray analyses were carried out using a glycosaminoglycan (GAG)-focused array based the neoglycolipid (NGL) technology ^18, 19^. The preparation of the GAG NGL probes, their formulation as liposomes, and printing using a noncontact robotic arrayer Nano-Plotter 2.1 (GeSim, Germany) onto 16 pad nitrocellulose-coated glass slides (Sartorius, Germany) at 2 and 5 fmol per spot in duplicate were as described previously ^18^. After blocking the slides for 1 h with 2% (w/v) bovine serum albumin (BSA; Merck A7030) in HBS buffer (10 mM HEPES, pH 7.4, 150 mM NaCl) containing 5 mM CaCl _2_, the microarrays were overlaid with sTMEM2 at 300 µg/mL for 90 minutes, followed by 1 h incubation with the detection antibody solution composed of mouse monoclonal anti-polyhistidine antibody (Merck SAB4200620) and the biotinylated goat anti-mouse IgG (Merck B7264) precomplexed at a 1:1 ratio and diluted in at 10 µg/mL, diluent: 1% (w/v) BSA in HBS containing 5 mM CaCl _2_. Binding was detected with Alexa Fluor-647-labelled streptavidin (Molecular Probes) at 1 µg/mL for 30 minutes. The control HA-binding protein was a native protein isolated from bovine nasal cartilage (complex of aggrecan G1 and link protein; Merck 385911), which was overlaid at 50 µg/mL followed by single step detection with Alexa Fluor-647-labelled streptavidin as mentioned above. The microarray images were recorded using a GenePix 4300A scanner (Molecular Devices).

## Results

### Crystal structure of sTMEM2

We expressed the soluble ectodomain of human TMEM2 (residues 106-1383; sTMEM2) with a N-terminal hexahistidine tag in Expi293 cells. We obtained a monodisperse glycoprotein that formed single crystals readily. We collected a high-redundancy X-ray diffraction data set (deposited in the PDB, see *Underlying data*) to 3.5 Å resolution and solved the structure by molecular replacement using the AlphaFold model of TMEM2 ^9^. After rebuilding residues 812-824 and adding N-linked glycans, we refined the structure conservatively to *R*_free_ = 0.319. Nearly the entire ectodomain (residues 112-1381) is resolved in the crystal structure, which matches the AlphaFold model with a root-mean-square (r.m.s.) deviation of only 1.1 Å (1270 Cα atoms). Thus, we have experimentally confirmed the remarkably accurate prediction of this multidomain protein.

**Figure 1.**
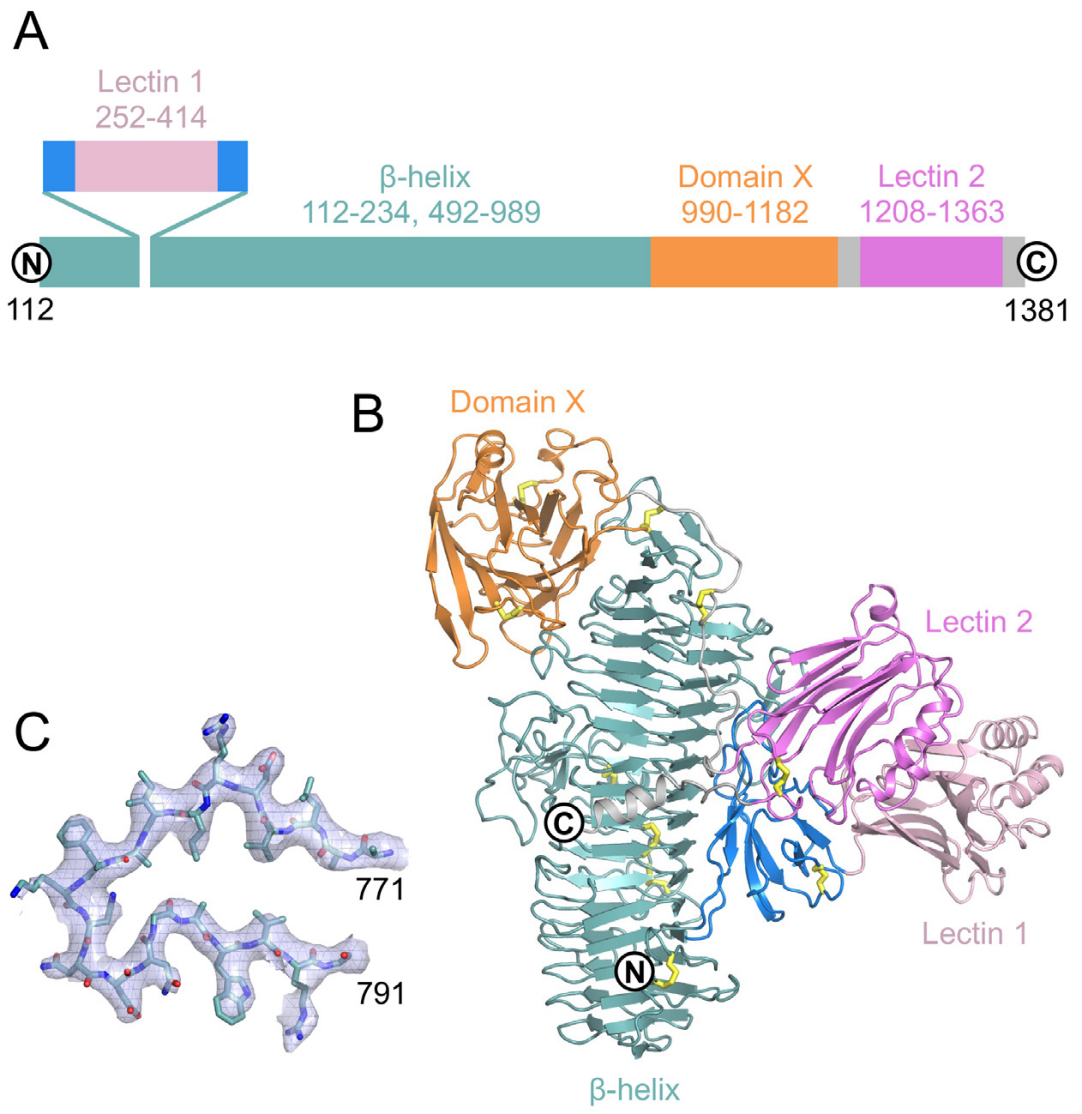
Crystal structure of soluble Transmembrane protein 2 (sTMEM2). ( **A**) Schematic representation of the sTMEM2 domain structure. Sequence numbers refer to full-length TMEM2. ( **B**) Cartoon drawing of the sTMEM2 structure using the same colour code as in A. Disulfide bonds are indicated by yellow sticks. *N*-linked glycans have been omitted for clarity. ( **C**) Electron density map for residues 771-791. Shown is the final 2 *F*_obs_- *F*_calc_ map contoured at 1 σ level. This figure was produced using PyMOL version 2.5.2. UCSF Chimera is a free alternative to PYMOL.

The sTMEM2 structure is built around a 70 Å-long right-handed parallel β-helix that starts at the N terminus ( Figure 1). The predicted G8 domain ^11^ is part of this continuous β-helix. A small β-sandwich and a larger lectin-like domain (26% sequence identity to the N-terminal domain of protein O-linked-mannose β-1,2-N-acetylglucosaminyltransferase 1 (POMGNT1) ^20^) are inserted into the β-helix after three complete turns without disrupting its regular structure. The β-helix is followed by a large β-sandwich that is not closely related to any other known structure (termed domain X). The polypeptide chain then runs halfway down the β-helix before forming a second lectin-like domain (20% identity to POMGNT1) and, finally, a C terminal helix. The two lectin-like domains interact through a modest interface, and together form a bulky addition to the central β-helix. The longest dimension of sTMEM2, from the tip of lectin-like domain 1 to domain X, is over 110 Å.

**Figure 2.**
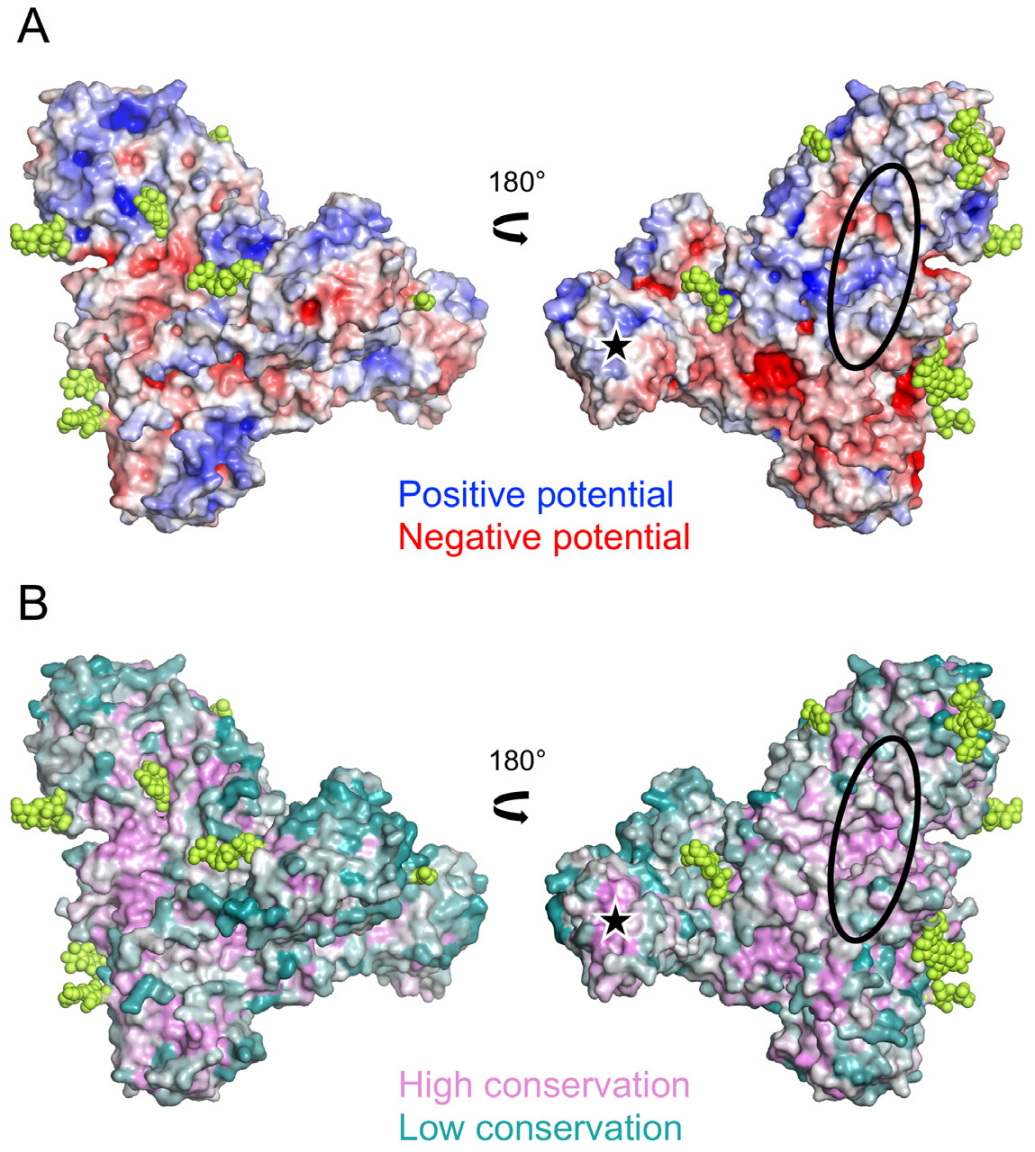
Surface properties of soluble Transmembrane protein 2 (sTMEM2). ( **A**) Electrostatic surface potential calculated using the APBS function of PyMOL (version 2.4.1). UCSF Chimera is a free alternative to PYMOL. Positive and negative potential is shown in blue and red, respectively. Glycans are shown in lime green. ( **B**) Sequence conservation calculated using the Consurf server ^22^. High and low sequence conservation is shown in magenta and teal, respectively. Glycans are shown in lime green. In A and B, the views on the left are the same as in Figure 1B. The black oval indicates the general location of active sites in related β-helix enzymes (see text). The black star indicates the tip of lectin-like domain 1.

sTMEM2 contains 24 cysteine residues, 20 of which are involved in disulphide bonds. Cys361, Cys554, Cys750, and Cys940 are unpaired and too distant from each other to plausibly form disulphide bonds. sTMEM2 contains 15 sequons for N-linked glycosylation, nine of which showed extra electron density for glycans in our structure. The electrostatic surface potential of sTMEM2 ( Figure 2A) shows a relatively even distribution of positive and negative potential, and no extended basic (positive) surface regions as may have been expected for a protein interacting with polyanionic HA. Sequence conservation is highest in the β-helix, with another notable region of high sequence conservation at the tip of lectin-like domain 1 ( Figure 2B).

### Comparison to other structures

**Figure 3.**
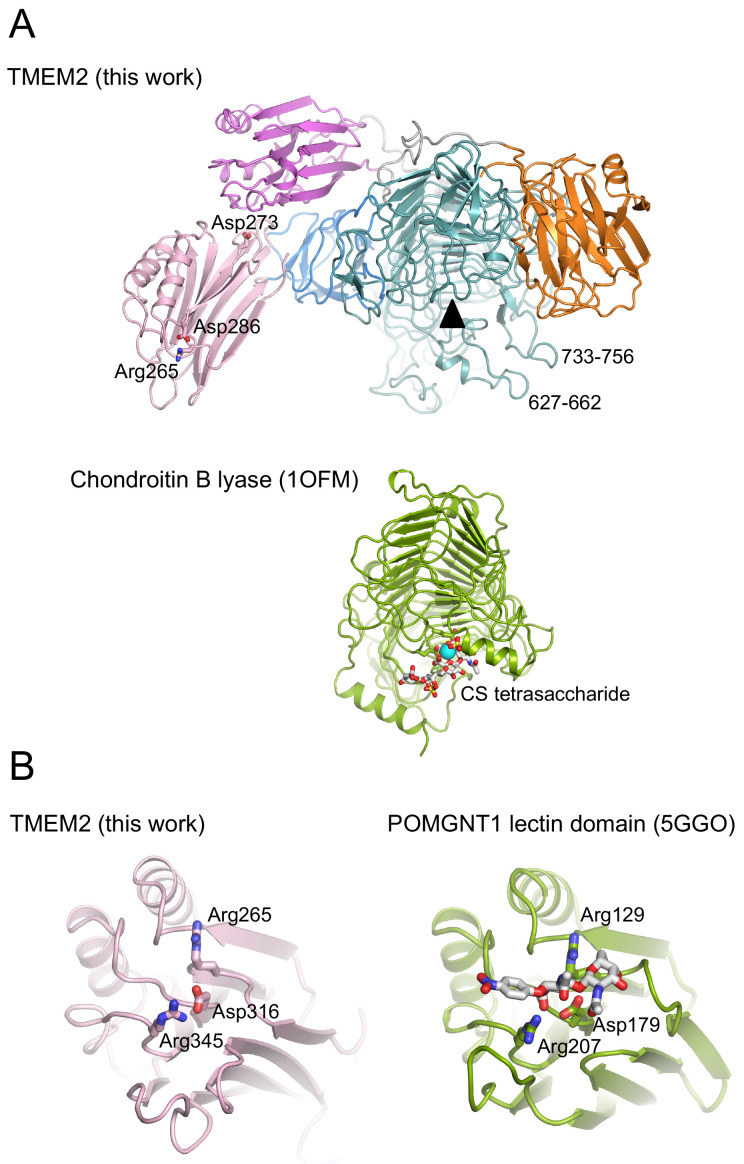
Comparison of soluble Transmembrane protein 2 (sTMEM2) to other proteins. ( **A**) Chondroitin lyase ( PDB 1OFM) was superimposed onto the β-helix of sTMEM2 with a r.m.s. deviation of 3.3 Å for 325 Cα atoms. Chondroitin lyase contains a Ca ^2+^ ion in the active site; the equivalent position in sTMEM2 is indicated by the black triangle. Three residues in the lectin-like domain 1 whose mutation has been reported to reduce TMEM2 activity ^5^ are shown in atomic detail. ( **B**) The N-terminal lectin domain of POMGNT1 ( PDB 5GGO) was superimposed onto the lectin-like domain 1 of sTMEM2 with a r.m.s. deviation of 2.3 Å for 153 Cα atoms. The disaccharide bound to POMGNT1 is shown in atomic detail, as are selected residues that are conserved between sTMEM2 and POMGNT1. This figure was produced using PyMOL version 2.5.2. UCSF Chimera is a free alternative to PYMOL.

The β-helix is a common structural motif of enzymes involved in polysaccharide degradation, encompassing both hydrolases (using water to break the glycosidic bond) and lyases (using a β-elimination mechanism). The latter family is restricted to enzymes acting on uronic polysaccharide substrates, such as HA and chondroitin sulphate ^21^. We used the DALI server ^16^ to identify structurally similar β-helix enzymes and mapped all active sites for which experimental confirmation was available (bound substrate and/or mutagenesis). Active sites are invariably located on the concave face of the β-helix ( Figure 2). The majority of active sites are located roughly in the middle of the β-helix, which in sTMEM2 is obstructed by two long loops, made up of residues 627-662 and 733-756 ( Figure 3A). A smaller number of β-helix enzymes have their active sites closer to the C-terminus of the β-helix (i.e. nearer the top in the view of Figure 2). The most relevant example is the *Pedobacter heparinus* chondroitin B lyase structure, which can be superimposed onto sTMEM2 with a r.m.s. deviation of 3.3 Å for 325 Cα atoms (8% sequence identity). The active site of chondroitin B lyase features a catalytic Ca ^2+^ ion bound by two acidic residues, Glu243 and Glu254 ( Figure 3A) ^23^. sTMEM2 contains a similarly sized cleft, but only one of the acidic residues is conserved, and there are no other suitable Ca ^2+^ ligands in the vicinity. Given that our biochemical experiments failed to detect any hyaluronidase activity of sTMEM2 (see below), the location of the putative active site on TMEM2 remains an open question.

As already mentioned, sTMEM2 contains two lectin-like domains, one inserted into the N-terminal region of the β-helix and another one at the very C-terminus. The closest homologue of known structure is the N-terminal domain of the glycosyltransferase POMGNT1, which binds β-linked GlcNAc ^20^. The three key sugar-binding residues of POMGNT1 are conserved in TMEM2, suggesting that lectin-like domain 1 may bind carbohydrates ( Figure 3B). It is also interesting that all of the substitutions that have been reported to abrogate TMEM2 activity ^5^ map to lectin-like domain 1: R265C, D273N, D286N. None of the three sugar-binding residues is conserved in lectin-like domain 2 of sTMEM2, suggesting that this domain does not bind carbohydrates.

### Hyaluronidase activity of sTMEM2

**Figure 4.**
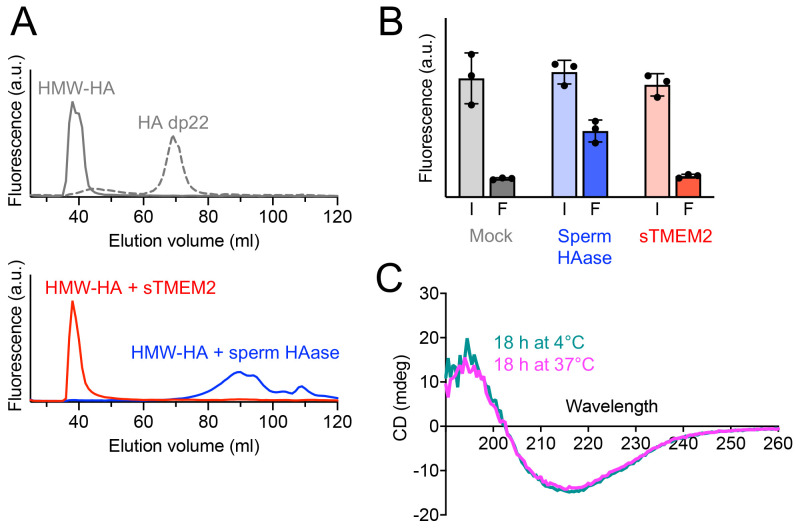
Hyaluronidase activity of soluble Transmembrane protein 2 (sTMEM2). ( **A**) Top, size exclusion chromatography of 100 μg/ml fluorescein-labelled high-molecular-weight Hyaluronic acid (HMW-HA) or HA 22-mer (dp22, ~5 kDa). Bottom; size exclusion chromatography of 100 μg/ml fluorescein-labelled HMW-HA incubated overnight with 100 μg/ml sTMEM2 or sperm hyaluronidase (pH 6.0, 1 mM CaCl _2_). Only the sperm hyaluronidase was able to degrade HMW-HA. Shown is a representative of *n* = 3 experiments. ( **B**) Ultrafiltration of 0.1 μg/ml fluorescein-labelled HMW-HA incubated overnight with 100 μg/ml sTMEM2 and sperm hylauronidase (pH 6.0, 1 mM CaCl _2_). The fluorescence was measured in the solution before and after filtration (I, input; F, filtrate). Only the sperm hyaluronidase was able to degrade HMW-HA. Data are presented as mean values ± s.e.m. for *n* = 3 independent experiments. ( **C**) Circular dichroism spectra of sTMEM2 incubated overnight at 4°C (teal) and 37°C (magenta). The minimum at 217 nm is characteristic of β-sheet structure. There is no evidence of denaturation at the higher temperature. The raw measurements are available in *Underlying data*.

As a first step towards identifying catalytic residues in sTMEM2, we established the enzymatic activity assay used by Yamamoto *et al*. ^5^ for TMEM2 and by two independent groups for CEMIP ^24, 25^. Fluorescently labelled high-molecular-weight (HMW) HA was incubated with sTMEM2 or hyaluronidase from bovine testes under the reported optimum conditions for sTMEM2 (pH 6.0, 1 mM CaCl _2_) ^5^. Size-fractionation of the reaction products revealed complete degradation of HMW-HA by the sperm hyaluronidase, but to our surprise we could detect no degradation of HMW-HA by sTMEM2 ( Figure 4A). We also tested other sTMEM2:HA ratios, but failed to detect any hyaluronidase activity of TMEM2 in this assay (the column we used would have separated fragments <80 kDa from intact HMW-HA). We then carried out experiments at the very low HMW-HA concentration of 0.1 μg/ml, using ultrafiltration to separate intact and degraded HA, but again failed to observe HMW-HA degradation by sTMEM2 ( Figure 4B). To exclude the possibility that sTMEM2 may not be stable at 37°C, we recorded circular dichroism spectra of protein samples incubated overnight at 4°C and 37°C ( Figure 4C). We observed no differences and concluded that unfolding or aggregation of sTMEM2 during the overnight incubation cannot be the reason for the failure to detect hyaluronidase activity. The data associated with Figure 4 are available in *Underlying data*.

We estimated an upper limit of k _cat_ as follows. In our experimental setup, an enzyme that degrades 0.1 μg/ml HMW-HA (molecular mass, 1 MDa) into 5 kDa fragments ^5^ would have to cleave 20 nM glycosidic bonds over the course of 18 hours, or 0.019 nM bonds per minute. The sTMEM2 concentration of 100 μg/ml corresponds to 700 nM putative enzyme. Thus, a k _cat_ of 0.019/700 = 2.6 x 10 ^-5^ min ^-1^ would be sufficient to observe complete degradation of the HMW-HA into 5 kDa fragments. For comparison, the k _cat_ value of human HYAL1 is 350 min ^-1^ (calculated from the v _max_ value reported in ref 26). Therefore, if sTMEM2 indeed has hyaluronidase activity, it is >10 ^7^-fold less active than a conventional HYAL enzyme.

**Figure 5.**
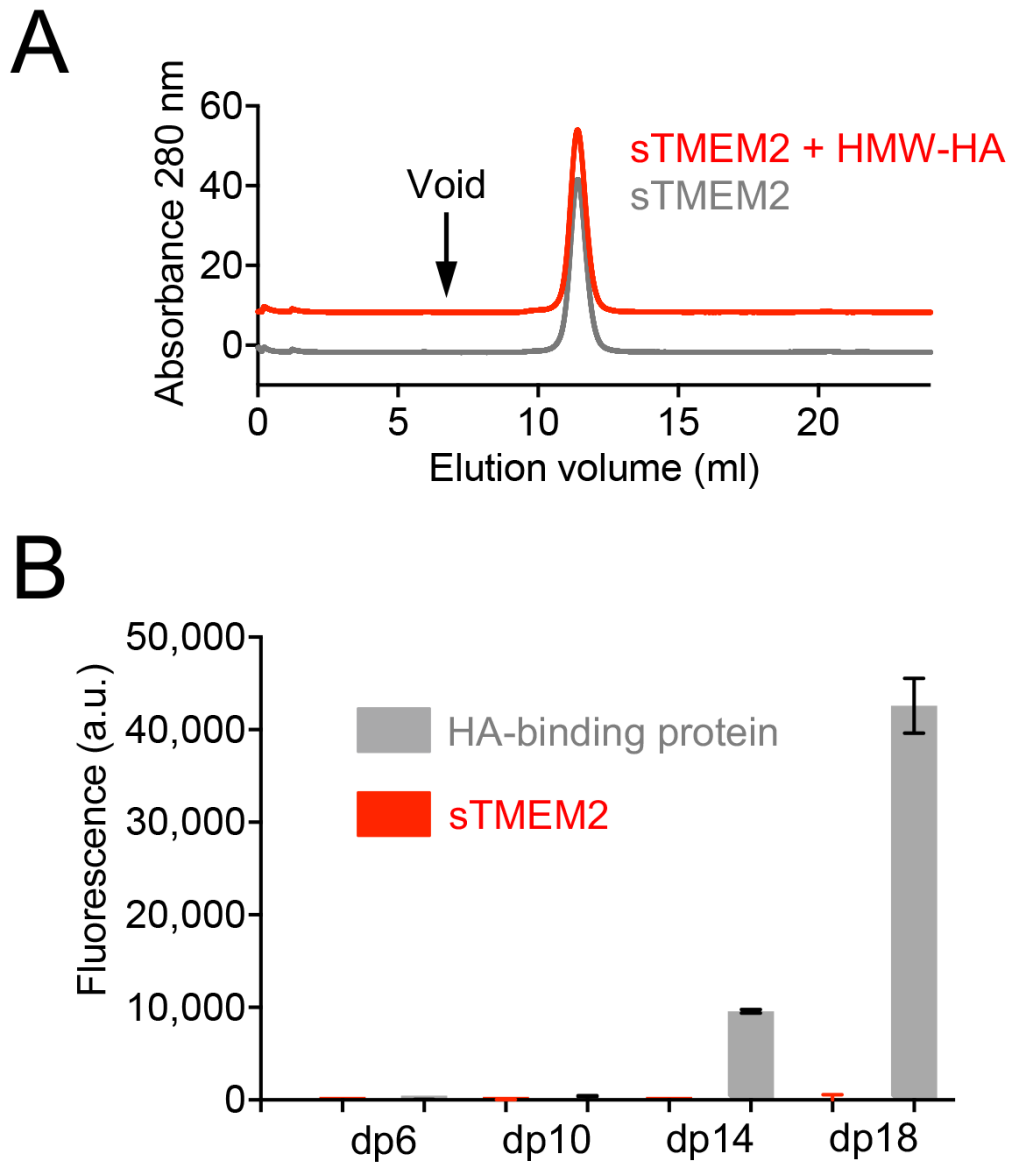
Hyaluronan binding by soluble Transmembrane protein 2 (sTMEM2). ( **A**) Size exclusion chromatography of sTMEM2 in the absence or presence of unlabelled HMW-HA. To avoid overlap of the two traces, the red trace has been shifted by +10 mAU. No sTMEM2 protein is associated with HMW-HA eluting in the void volume of the column (arrow). ( **B**) Binding of sTMEM2 and the aggrecan G1-link protein complex (HA-binding protein) to HA oligomers of different length (dp, degree of polymerisation). The experiment was done using a neoglycolipid-based microarray ^18, 19^. Data (fluorescence intensities of HA probes arrayed at 5 fmol/spot level) are presented as mean values ± range for *n* = 2 duplicate spots on the array. The raw measurements are available in *Underlying data*.

Next, we tested whether sTMEM2 interacts with HMW-HA in solution. sTMEM2 eluted in a sharp symmetrical peak at 11.4 ml from a Superdex S200 increase 10/300 column regardless of whether it had been pre-incubated with HMW-HA or not ( Figure 5a). Any sTMEM2 associated with HMW-HA would have eluted either in the void volume of the column (stable complex) or between the void volume and the elution volume of free sTMEM2 (complex dissociating during chromatography). We also tested HA binding using a glycosaminoglycan-focused microarray ^18^. sTMEM2 did not bind to any of the HA probes on this array, whereas a known HA-binding protein (aggrecan G1-link protein complex) showed robust binding to oligosaccharides longer than 10 sugar residues ( Figure 5B). We conclude that if sTMEM2 interacts with HA, it does so only weakly. We note that the *K*_M_ value of sperm hyaluronidase for a HA octasaccharide is 130 μM ^27^. A similarly low affinity of sTMEM2 for HA could explain why we did not observe an interaction in two different assay formats. The data associated with Figure 5 are available in *Underlying data*. 

## Discussion

We found that pure, crystallisable, soluble TMEM2 has no detectable hyaluronidase activity. Two possibilities need to be considered: (1) TMEM2 is not a hyaluronidase, contrary to what has been reported ^5^; or (2) TMEM2 is a hyaluronidase, but activity is lost when the ectodomain is severed from the plasma cell membrane. With regards to the former possibility, we are not the first ones to observe negligible HA degradation by TMEM2. Knock-down experiments in human skin fibroblasts ^24^ and chondrocytes from osteoartritic knee cartilage ^25^ demonstrated involvement of CEMIP but not of TMEM2 in HA degradation. Moreover, hyaluronidase activity in skin homogenates was found to be robust at the acidic pH optimum of HYALs, but negligible at the neutral pH optimum reported for TMEM2, even though TMEM2 is highly expressed in skin ^28^. How these negative results may be reconciled with reports that TMEM2 is an extracellular hyaluronidase essential for systemic HA turnover ^5, 6^ is a matter for future studies. Other functions ascribed to TMEM2, such as its roles in development ^29– 31^ and endoplasmic reticulum homeostasis ^8^, do not require TMEM2 itself to be a hyaluronidase. For instance, TMEM2 may be a component of the machinery that translocates HA into the cell for eventual lysosomal degradation. The second possibility is that TMEM2 hyaluronidase activity requires localisation of the protein at the plasma membrane, either because of some steric reason or because of the presence of an essential protein partner or cofactor. This situation would be akin to what has been reported for CEMIP, which appears to be active only in clathrin-coated pits ^4^. Clustering of TMEM2 at the cell surface, which would greatly amplify its avidity for HA, may be required for HA degradation. Whatever the reason, our finding that pure sTMEM2 is at least 10 ^7^-fold less active than conventional HYALs complicates further enzymological studies.

What can be learned from the sTMEM2 structure? First of all, it dramatically highlights the power of AlphaFold in predicting complex multi-domain structures ^9^. Second, it allows a better interpretation of previous mutagenesis experiments. Mutation of Arg187 in CEMIP and its equivalent Arg265 in TMEM2 reduced HA degradation ^4, 5^. Arg265 is located at the tip of lectin-like domain 1, in an equivalent position as a critical sugar-binding residue in a related domain ( Figure 3). It is unlikely that Arg265 participates in catalysis, but it may bind HA weakly and present it to an active site elsewhere on TMEM2 or an associated protein. Such a “molecular ruler” effect could explain why TMEM2 products are ~5 kDa fragments and not ~400 Da disaccharides as in the case of HYALs ^5^. Finally, the finding that the G8 domain is actually part of the β-helix explains why previous attempts to functionally dissect the TMEM2 ectodomain were unsuccessful ^30^.

## Data Availability

Protein Data Bank: Structure factors and coordinates of the sTMEM2 structure. Accession number 8C6I; https://www.rcsb.org/structure/8C6I. Figshare: Hyaluronidase activity of sTMEM2 (SEC). https://doi.org/10.6084/m9.figshare.21975914.v2. This project contains the following underlying data: Hyaluronidase activity of sTMEM2 (SEC). Figshare: Hyaluronidase activity of sTMEM2 (Filtration). https://doi.org/10.6084/m9.figshare.21975920.v2. This project contains the following underlying data: Hyaluronidase activity of sTMEM2 (Filtration). Figshare: Circular dichroism spectra of sTMEM2. https://doi.org/10.6084/m9.figshare.22548601 This project contains the following underlying data: Circular dichroism spectra of sTMEM2. Figshare: Hyaluronan binding of sTMEM2 (SEC). https://doi.org/10.6084/m9.figshare.21975917.v2. This project contains the following underlying data: Hyaluronan binding of sTMEM2 (SEC). Figshare: Hyaluronan binding of sTMEM2 (Microarray). https://doi.org/10.6084/m9.figshare.22434085 This project contains the following underlying data: Hyaluronan binding of sTMEM2 (Microarray). Data are available under the terms of the Creative Commons Attribution 4.0 International license (CC-BY 4.0).
