# Supplementary material for: Structure of the transmembrane protein 2 (TMEM2) ectodomain and its apparent lack of hyaluronidase activity
Source: Wellcome Open Res. 2023 May 2;8:76. Originally published 2023 Feb 14. [Version 2] doi: 10.12688/wellcomeopenres.18937.2 (PMC10206443; doi:10.12688/wellcomeopenres.18937.2)
Supplement: Peer review history

# Peer review: Reviewer response for version 2

**DOI:** 10.21956/wellcomeopenres.21506.r56861

**Published:** 2023-05-23

**Authors:** Kristina Nesporova

**Affiliations:** 1 R&D Department, Contipro a.s, Dolni Dobrouc, Czech Republic

The revised version (v2) of the article sufficiently addresses previous shortcomings.

Is the work clearly and accurately presented and does it cite the current literature?

Yes

If applicable, is the statistical analysis and its interpretation appropriate?

Not applicable

Are all the source data underlying the results available to ensure full reproducibility?

Partly

Is the study design appropriate and is the work technically sound?

Partly

Are the conclusions drawn adequately supported by the results?

Partly

Are sufficient details of methods and analysis provided to allow replication by others?

Partly

Reviewer Expertise:

hyaluronan structure and hyaluronan/protein interactions, hyaluronidase activity assessment

I confirm that I have read this submission and believe that I have an appropriate level of expertise to confirm that it is of an acceptable scientific standard.

# Peer review: Reviewer response for version 2

**DOI:** 10.21956/wellcomeopenres.21506.r56862

**Published:** 2023-05-15

**Authors:** Rebekka Wild

**Affiliations:** 1 Institut de Biologie Structurale, UMR 5075, CNRS, CEA, University Grenoble Alpes, Grenoble, France

The revised version (v2) of the article sufficiently addresses previous shortcomings.

Is the work clearly and accurately presented and does it cite the current literature?

Yes

If applicable, is the statistical analysis and its interpretation appropriate?

Yes

Are all the source data underlying the results available to ensure full reproducibility?

Yes

Is the study design appropriate and is the work technically sound?

Partly

Are the conclusions drawn adequately supported by the results?

Partly

Are sufficient details of methods and analysis provided to allow replication by others?

Yes

Reviewer Expertise:

Glycosaminoglycan biosynthesis, structural biology, glycobiology, cryo-EM

I confirm that I have read this submission and believe that I have an appropriate level of expertise to confirm that it is of an acceptable scientific standard.

# Peer review: Reviewer response for version 1

**DOI:** 10.21956/wellcomeopenres.20995.r55041

**Published:** 2023-03-01

**Authors:** Kristina Nesporova

**Affiliations:** 1 R&D Department, Contipro a.s, Dolni Dobrouc, Czech Republic

The manuscript by Niu et al. deals with the interesting topic of structural and biochemical analysis of relatively novel hyaluronidase TMEM2. Authors are trying to broaden the knowledge about this protein which seems to play an important role in physiological and pathological processes in various tissues.

 The first (and possibly the main) part of this work is focused on the structure of the soluble ectodomain of TMEM2 which should be responsible for hyaluronan degradation. The second part of this study then deals with the biochemical/enzymological measurements. The overall study is rather brief and straightforward but it doesn't necessarily lessen its impact.

 I don’t find any obvious problems with the structural study and it seems that the very high correlation of AlphaFold prediction with the actual crystal structure is a good indicator of the reliability of these results. It is also important to see this high level of correlations which might promote using this in silico method. As my area of expertise is more in hyaluronidase activity detection, I will focus more on the second part of the study.

 The method used for hyaluronidase activity assessment has been already used by Yamamoto et al. and for well-characterized samples of hyaluronidases I don’t doubt its usefulness. For a partial protein whose optimum for enzymatic activity is not well known, I would recommend alternative methods which don’t rely on the labeled HA as the chemical modification can affect the degradation process. Such a method can be agarose electrophoresis with subsequent HA staining or more quantitative analytical methods such as SECMALS or even LC-MS for small fragment identification and quantification. When using the fluorescently labeled substrate, at least the degree of substation should be included in the substrate description in the method section. And I would recommend the analyzed the content of free fluorophore molecules, as those are often present. Those can be eliminated by an ultracentrifugation filter with a relatively low cut-off (only low molecular fluorescein and other possible residues from the chemical reaction will be filtered off). As you used the mock protein with no hyaluronidase activity the possible contamination with free fluorescein can be evaluated, but using pure labeled HMW HA is always preferable.

 I am also missing information about the enzymatic activity of sperm hyaluronidase used as a positive control. As the term sperm hyaluronidase is used broadly for various preparation of PH20 (various animal origin, various purity of preparations) the activity is more important information than the concentration (add the supplier or manufacturer of BTH used and its activity). I am also rather surprised that the sperm hyaluronidase at the 100 ug/ml concentration during the 18h degradation protocol hasn’t degraded the HA fully (Fig 4B). But without the information about BTH activity, I cannot judge this. Even without the activity info, I would like to ask the authors to explain why the increase of LWM HA fragment in the sperm hyaluronidase sample (Fig 4B) is not accompanied by a decrease in HMW HA.

 I would be also very careful with the conclusion from very preliminary results using the SEC for the detection of the sTMEM2/HA complexes. The composition of the mobile phase can drive the formation and disruption of noncovalent complexes of enzymes and substrates. Would the same method enable us to see the hyaluronidase/HA complexes formation with the control sperm hyaluronidase?

 The section of the article dealing with enzymatic activity provides mostly preliminary data. I appreciate that the authors are not making any major conclusions from them but the final version of this manuscript would benefit from the addition of some optimization steps or better discussion of obtained results from activity assays.

Is the work clearly and accurately presented and does it cite the current literature?

Yes

If applicable, is the statistical analysis and its interpretation appropriate?

Not applicable

Are all the source data underlying the results available to ensure full reproducibility?

Partly

Is the study design appropriate and is the work technically sound?

Partly

Are the conclusions drawn adequately supported by the results?

Partly

Are sufficient details of methods and analysis provided to allow replication by others?

Partly

Reviewer Expertise:

hyaluronan structure and hyaluronan/protein interactions, hyaluronidase activity assessment

I confirm that I have read this submission and believe that I have an appropriate level of expertise to confirm that it is of an acceptable scientific standard, however I have significant reservations, as outlined above.

# Response

**Published:** 2023-04-20

**Authors:** Erhard Hohenester

**Affiliations:** Imperial College London, UK

1. " *The method used for hyaluronidase activity assessment has been already used by Yamamoto et al. and for well-characterized samples of hyaluronidases I don’t doubt its usefulness. For a partial protein whose optimum for enzymatic activity is not well known, I would recommend alternative methods which don’t rely on the labeled HA as the chemical modification can affect the degradation process. Such a method can be agarose electrophoresis with subsequent HA staining or more quantitative analytical methods such as SECMALS or even LC-MS for small fragment identification and quantification."*
**We refer to our response to reviewer 1 (point 4). The assay has been used not only by Yamamoto et al., but also by two other groups to show hyaluronidase activity of cell-associated CEMIP (references 23 and 24 of the revised manuscript). We therefore feel that this is a validated assay for this class of proteins.  **
2. *"When using the fluorescently labeled substrate, at least the degree of substation should be included in the substrate description in the method section."*
**The degree of substitution is ~5%. This information has been added to the revised manuscript.  **
3. *"And I would recommend the analyzed the content of free fluorophore molecules, as those are often present. Those can be eliminated by an ultracentrifugation filter with a relatively low cut-off (only low molecular fluorescein and other possible residues from the chemical reaction will be filtered off). As you used the mock protein with no hyaluronidase activity the possible contamination with free fluorescein can be evaluated, but using pure labeled HMW HA is always preferable."*
**We would have detected any free fluorophore during size exclusion experiments. There is no fluorescence signal between 50 and 120 ml (the column volume).  **
4. *"I am also missing information about the enzymatic activity of sperm hyaluronidase used as a positive control. As the term sperm hyaluronidase is used broadly for various preparation of PH20 (various animal origin, various purity of preparations) the activity is more important information than the concentration (add the supplier or manufacturer of BTH used and its activity)"*
**The supplier and catalogue number of the sperm hyaluronidase was given in the original manuscript (Methods). We have added information on the specific activity, as requested.  **
5. *"I am also rather surprised that the sperm hyaluronidase at the 100 ug/ml concentration during the 18h degradation protocol hasn’t degraded the HA fully (Fig 4B). But without the information about BTH activity, I cannot judge this. Even without the activity info, I would like to ask the authors to explain why the increase of LWM HA fragment in the sperm hyaluronidase sample (Fig 4B) is not accompanied by a decrease in HMW HA."*
There seems to be a misunderstanding here. Figure 4A shows that the sperm hyaluronidase is able to fully degrade 100 ug/ml HMW-HA (complete disappearance of the void peak). The experiment in Figure 4B used a 1000-fold lower HMW-HA concentration. The bars labelled “I” do not show the HMW-HA remaining *after* incubation with protein/enzyme. Rather, they show fluorescence *before* incubation, to demonstrate equal input in all experiments. Why not all input fluorescence is recovered in the filtrate is unclear. The device has a dead volume and some of the fluorescent molecules may have stuck to the ultrafiltration membrane.  
6. *"I would be also very careful with the conclusion from very preliminary results using the SEC for the detection of the sTMEM2/HA complexes. The composition of the mobile phase can drive the formation and disruption of noncovalent complexes of enzymes and substrates. Would the same method enable us to see the hyaluronidase/HA complexes formation with the control sperm hyaluronidase?"*
**The *K*_M_ value of sperm hyaluronidase for HA appears to be quite high (>100 μM), so it is not clear whether binding would be detected. A sentence on this point has been added to the revised manuscript. In response to a comment by reviewer 1, we tested glycan binding by sTMEM2 in a glycosaminoglycan-focused microarray. We observed no binding to any of the probes and have added the negative data for HA probes to the revised manuscript (new Figure 5B).**
7. *"The section of the article dealing with enzymatic activity provides mostly preliminary data. I appreciate that the authors are not making any major conclusions from them but the final version of this manuscript would benefit from the addition of some optimization steps or better discussion of obtained results from activity assays."*
We refer to our response to reviewer 1 (point 4). We are confident that our sTMEM2 protein is not detectably active under the optimum conditions reported by Yamamoto et al. (2017). We feel that this is a valuable observation that should be in the public domain. Of course, we cannot categorically exclude that sTMEM2 has hyaluronidase activity under certain, untested, conditions. We believe we have been careful in the discussion of our negative finding. We concede that the original title did not convey our uncertainty well enough, and we have therefore changed it to *apparent* lack of hyaluronidase activity. We would like to add that, as a matter of courtesy, we informed Dr Yamaguchi of our negative finding a year before we submitted the study for publication.

# Peer review: Reviewer response for version 1

**DOI:** 10.21956/wellcomeopenres.20995.r55040

**Published:** 2023-03-01

**Authors:** Rebekka Wild

**Affiliations:** 1 Institut de Biologie Structurale, UMR 5075, CNRS, CEA, University Grenoble Alpes, Grenoble, France

In their article, Niu et al. describe the crystal structure of TMEM2, a cell surface protein previously reported to have hyaluronidase activity. Hyaluronic acid (HA) is a long, linear polysaccharide with diverse biological functions and its regulated degradation helps to fine-tune its activity. The described structure at 3.5 Å resolution reveals that TMEM2 folds into a large β-helix and three globular domain. Structural similarities of the β-helix with the chondroitin B lyase from Pedobacter heparinus indicates a potential catalytic site in the concave face of the β-helix. Similarities between the lectin 1 domain and the N-terminal domain of POMGNT1, involved in N-acetylglucosamine binding, suggest a potential involvement in glycan recognition. Despite the structural similarities of TMEM2 with other enzymes involved in glycan metabolism, the purified TMEM2 does not show any detectable *in vitro* hyaluronidase activity and no binding of high-molecular weight hyaluronic acid to TMEM2 was observed in size-exclusion chromatography.

- The TMEM2 structure at 3.5 Å resolution was solved by molecular replacement using the AlphaFold predicted model. A panel illustrating the quality of the map (map around the model) would be helpful to assess the structure.
- A soluble, secreted form of TMEM2 was previously reported to have hyaluronidase activity (Yamamoto et al., JBC 2017). Using similar reaction conditions, Niu et al. don’t observe any hydrolysis of high-molecular weight HA (HMW-HA). This article raises the big question if TMEM2 has hyaluronidase activity or not. Although some possible explanations addressing the question why no activity is observed are provided by the authors, a more extensive analysis of this is needed (see experiments suggested below).
- A more comprehensive analysis of the structure with other glucosidases could help to pin-point conserved and distinct residues in a potential active site in more detail.
- The activity assay is carried out at 37°C for 18h, however, the thermal stability of purified TMEM2 is unknown. A thermal stability assay (nano differential scanning fluorimetry or others) would help to confirm that the chosen reaction conditions (T) are appropriate.
- A more extensive screening of different reaction conditions (pH range, metal ions,...) should be considered.
- A 800 kDa HMW-HA is used for enzymatic assays. Partial cleavage of this large polysaccharide would lead to products that could still elute in the void volume of the Sephacryl S200 HR column. It is not discussed in the article, what is the maximum length of generated fragments that is distinguishable from the uncleaved substrate.
- Only one substrate (800 kDa) HMW-HA was used to study potential TMEM2 activity. Additional activity assays using HA of different/shorter lengths could help to detect hyaluronidase activity.
- Binding of HMW-HA to TMEM2 was studied by size-exclusion chromatography. However, size exclusion chromatography is more useful to study high-affinity interactions rather than low affinity interactions. A second approach to measure TMEM2 interaction with HA or even another glycosaminoglycan could help to reveal lower affinity interactions.

Is the work clearly and accurately presented and does it cite the current literature?

Yes

If applicable, is the statistical analysis and its interpretation appropriate?

Yes

Are all the source data underlying the results available to ensure full reproducibility?

Yes

Is the study design appropriate and is the work technically sound?

Partly

Are the conclusions drawn adequately supported by the results?

Partly

Are sufficient details of methods and analysis provided to allow replication by others?

Yes

Reviewer Expertise:

Glycosaminoglycan biosynthesis, structural biology, glycobiology, cryo-EM

I confirm that I have read this submission and believe that I have an appropriate level of expertise to confirm that it is of an acceptable scientific standard, however I have significant reservations, as outlined above.

## References

[1] : A mammalian homolog of the zebrafish transmembrane protein 2 (TMEM2) is the long-sought-after cell-surface hyaluronidase. *J Biol Chem* .2017;292(18) : 10.1074/jbc.M116.770149 7304-7313 10.1074/jbc.M116.770149 28246172PMC5418033

# Response

**Published:** 2023-04-20

**Authors:** Erhard Hohenester

**Affiliations:** Imperial College London, UK

1. *"The TMEM2 structure at 3.5 Å resolution was solved by molecular replacement using the AlphaFold predicted model. A panel illustrating the quality of the map (map around the model) would be helpful to assess the structure."*
**A figure of a representative portion of the map has been added to Figure 1.  **
2. *"A more comprehensive analysis of the structure with other glucosidases could help to pin-point conserved and distinct residues in a potential active site in more detail."*
**Our analysis is now described in more detail. The majority of β-helix enzymes have their active sites in a region that is obstructed by two long loops in TMEM2 (now labelled in Figure 3A). Among the few enzymes that have their active sites in a more C-terminal location, chondroitinase B is the most relevant example in terms of the reaction catalysed and the dependence on calcium ions. Neither electrostatic potential nor sequence conservation are of help in locating a putative active site (revised Figure 2).  **
3. *"The activity assay is carried out at 37°C for 18h, however, the thermal stability of purified TMEM2 is unknown. A thermal stability assay (nano differential scanning fluorimetry or others) would help to confirm that the chosen reaction conditions (T) are appropriate."*
**We have measured circular dichroism spectra before and after incubation at 37°C for 18 hours and found no evidence of thermal instability. These data are now presented in a revised Figure 4.  **
4. " *A more extensive screening of different reaction conditions (pH range, metal ions,...) should be considered."*
We measured activity under the reported optimum conditions for sTMEM2 (Yamamoto et al., 2017). It is important to note that the assay with fluorescently labelled HMW-HA has been used not only by Yamamoto et al., but also by two other groups who showed hyaluronidase activity of cell-associated CEMIP (references 23 and 24 of the revised manuscript). Our experiments include a positive control and we now have experimental verification that our sTMEM2 protein is thermally stable. Therefore, we are confident that our sTMEM2 protein is not detectably active under the reported optimum conditions. We feel that this is a valuable observation that should be in the public domain. Of course, we cannot categorically exclude that sTMEM2 has hyaluronidase activity under certain, untested, conditions. We believe we have been careful in the discussion of our negative finding. We concede that the original title did not convey our uncertainty well enough, and we have therefore changed it to *apparent* lack of hyaluronidase activity. We would like to add that, as a matter of courtesy, we informed Dr Yamaguchi of our negative finding  a year before we submitted the study for publication.  
5. *"A 800 kDa HMW-HA is used for enzymatic assays. Partial cleavage of this large polysaccharide would lead to products that could still elute in the void volume of the Sephacryl S200 HR column. It is not discussed in the article, what is the maximum length of generated fragments that is distinguishable from the uncleaved substrate."*
**The S200 column would have separated fragments as large as 80 kDa from the void peak. This information has been added to the text.  **
6. *"Only one substrate (800 kDa) HMW-HA was used to study potential TMEM2 activity. Additional activity assays using HA of different/shorter lengths could help to detect hyaluronidase activity."*
**See our reponse to point 3. We used a substrate that was degraded by cell-associated CEMIP in two independent studies using the same assay.  **
7. *"Binding of HMW-HA to TMEM2 was studied by size-exclusion chromatography. However, size exclusion chromatography is more useful to study high-affinity interactions rather than low affinity interactions. A second approach to measure TMEM2 interaction with HA or even another glycosaminoglycan could help to reveal lower affinity interactions."*
**In response to this comment, we tested binding of sTMEM2 to a glycosaminoglycan-focused microarray. We observed no binding to any of the probes and have added the negative data for HA probes to the revised manuscript (new Figure 5B). We have also added a line to the discussion that the high avidity at cell-matrix contact sites may allow HA binding and degradation.**
